# Onwards! Open science and the (PLOS) genetics community

**DOI:** 10.1371/journal.pgen.1011466

**Published:** 2024-11-28

**Authors:** Aimée M. Dudley, Anne Goriely, Julia Squarr

**Affiliations:** 1 Pacific Northwest Research Institute, Seattle, Washington, United States of America; 2 MRC Weatherall Institute of Molecular Medicine, Radcliffe Department of Medicine, University of Oxford, Oxford, United Kingdom; 3 PLOS GmbH, Berlin, Germany; Harvard Medical School, UNITED STATES OF AMERICA

Much has changed since 2003 when the Public Library of Science (PLOS) spearheaded the groundbreaking model of open-access publishing that has reshaped the publishing industry. PLOS’s vision has always focused on open science and community engagement, and as the first “community journal” in the portfolio, *PLOS Genetics* has been at the forefront of this pioneering approach. As we step into our new roles as Editors-in-Chief, we would like to reflect on these aspirations and share some initial thoughts on how we hope to build on these important values in the years ahead.

To us, one of the most important qualities of *PLOS Genetics* is that it is, at its core, a community journal. The motto championed by the former Editors-in-Chief, Gregory Barsh and Gregory Copenhaver, emphasized that *PLOS Genetics* is a journal “by working scientists, for working scientists” [1–3]. As such, our mission extends far beyond simply publishing high-quality, peer-reviewed science. The journal seeks to provide the wider Genetics community with a platform for discussion and a unique opportunity for the “co-production” of science. In this way, practicing scientists are able to define and influence the *entirety* of the scientific process—from framing key scientific questions to setting the standards for how high-quality science is conducted and evaluated. It includes promoting the exchange of ideas and encouraging cross-disciplinary collaborations in a trusted, ethical, and collegial environment. We must also acknowledge the role of transparency and scientific consensus in driving meaningful progress. At the heart of this process are the *people* willing to respectfully engage in open debate in order to foster ethical research.

Being deeply embedded within the community also affords opportunities for training and mentoring the next generation of scientists, including future contributors to the journal. This is a priority for us because, now more than ever, *how* we conduct science matters. Those of us who have committed our working lives to science understand the importance of positive, equitable, diverse, and inclusive environments. We also recognize that different life experiences, perspectives, and backgrounds influence the questions we find interesting and important. Looking through diverse lenses enriches the scientific enterprise by including those who approach scientific problems in innovative ways. Since its inception, *PLOS Genetics* has published research that spans an unusually wide range of organisms, methodologies, and research topics. While this diversity in topics and approaches has been one of the journal’s greatest strengths, such breadth also brings practical challenges. *PLOS Genetics* benefits from a strong board of approximately 30 Section Editors and a devoted group of more than 200 Academic Editors, whose expertise spans the full range of organisms (from bacteria to humans) and covers a broad array of subjects (from fundamental and translational genetics to cross-disciplinary research that advances our understanding of biological principles). We are also supported by an incredibly dedicated team of PLOS professionals.

During our tenure, we will further engage and expand this large network of committed individuals, seeking to connect and extend our community of practicing scientists. We are especially committed to ensuring the future of Genetics research by bringing early-career researchers to the table, sharing good practices, and incorporating new voices and novel perspectives to the journal. We hope that by fostering an inclusive and supportive community, we can continue to promote fair, open, and creative science while expanding the boundaries of scientific discovery on a global scale. Additionally, we recognize that the field of Genetics evolves quickly, and we must ensure that the journal adapts accordingly.

We are excited to move forward in our shared mission to use genetics to tackle some of the world’s most pressing challenges in areas such as health, agriculture, and climate change. We will also keep at the forefront the essential role that breakthrough, high-quality, discovery-based science plays in driving this ambitious endeavor. We hope that you will be an integral part of this journey and will support us in our efforts to continue growing a vibrant community and journal that embraces diverse and inclusive approaches to science, discovery, and education.
